# An in vitro assessment of teething gels’ effects on human gingival mesenchymal stem cells

**DOI:** 10.1186/s12903-024-04213-w

**Published:** 2024-05-17

**Authors:** Sinem Birant, Sabiha Ceren İlisulu, Senem Kılıç, Zeynep Tunca, Hazal Özcan, Tunç Akkoç, Figen Seymen

**Affiliations:** 1 Faculty of Dentistry, Department of Pediatric Dentistry, Istanbul University-Cerrahpaşa, Istanbul, Turkey; 2 Faculty of Dentistry, Department of Pediatric Dentistry, Altınbaş University, Istanbul, Turkey; 3 Faculty of Medicine, Department of Immunology, Marmara University, Istanbul, Turkey; 4 Faculty of Medicine, Department of Pediatric Allergy-Immunology, Marmara University, Istanbul, Turkey

**Keywords:** Teething gel, Lidocain, Hyaluranic acid, Stem cell, Cell viability

## Abstract

**Background:**

The aim of this study is to examine the cytotoxic effects of dental gels with different contents, which are frequently used during teething, on gingival mesenchymal stem cells (G-MSCs).

**Method:**

The teething gels used in this study were Dentinox, Gengigel, Osanite, and Jack and Jill. The human gingival mesenchimal stem cells (hG-MSCs) were incubated with these teething gel solutions (0.1%, 50% and 80% concentrations). Reproductive behavior of G-MSCs was monitored in real time for 72 h using the xCELLigence real-time cell analyzer (RTCA) system. Two-way repeated Anova test and post hoc Bonferroni test were used to evaluate the effect of concentration and dental gel on 0-hour and 72-hour viability. Significance was evaluated at *p* < 0.05 level.

**Results:**

Teething gels prepared at 50% concentration are added to the G-MSC culture, the “cell index” value of G-MSCs to which Dentinox brand gel is added is significantly lower than all other groups (*p* = 0.05). There is a statistically significant difference between the concentrations in terms of cell index values at the 72nd hour compared to the 0th hour (*p* = 0.001).

**Conclusions:**

The local anesthetic dental gels used in children have a more negative effect on cell viability as concentration increases.

## Introduction

Tooth eruption is a normal developmental process that is defined as a set of events that includes the development of the tooth in the alveolar bone to its functional position in the jaw [1]. Genetic, molecular, cellular and textural factors are at the beginning of the factors that are effective in the realization of this event [2, 3]. In this natural process, many local and systemic symptoms can be seen in infants and young children. Local symptoms such as an increase in the amount of saliva experienced during this period, irritation in the gums, an increase in the desire to chew and bite, and systemic symptoms such as fever, diarrhea, vomiting, insomnia, restlessness, rashes in the body, and loss of appetite are often associated with this period [4–6].

Many pharmacological and non-pharmacological methods are used in the management of symptoms that occur during the eruption period. While pharmacological methods include the use of pain relievers, antipyretics and dental gels, non-pharmacological applications such as cold application, use of teether, massaging the gums are among the non-pharmacological applications [5–7]. Topical dental gels, one of the preferred methods among pharmacological applications, can be used to treat oral aphthae ulcers as well as teething problems, to provide anti-inflammatory effect in periodontal disorders and to accelerate healing in the post-oral surgery period [8].

Topical dental gels, which are frequently preferred during tooth eruption, have different contents. These gels can contain local anesthetics such as lidocaine, benzocaine, analgesic substances such as choline salicylate, substances found in the basic structure of the body such as hyaluronic acid, and herbal ingredients such as black mulberry, chamomile and clove extract [9].

Unconscious use of these gels, which are available without a prescription, during teething can cause serious side effects such as chemical burns, methemoglobinemia, allergies, and seizures [10, 11]. It has also been reported that gels containing topical anesthetics may cause iatrogenic oral mucosal trauma, tenderness or suffocation. It is stated that ingestion of these gels may also increase the risk of aspiration by numbing the mucous membranes of the child [8]. In addition, in 2011, the FDA issued a warning against the use of gels containing benzocaine due to the risk of methemoglobin [12]. Teething gels containing lidocaine can cause problems such as paresthesia, hypotension, seizures, bradycardia and cardiac arrest [10, 11].

Additionally, Fedder et al. stated that local anesthetics containing lidocaine, bupivacaine and ropivacaine have a cytotoxic effect on fibroblasts [13]. Contrary to these studies, it has been reported that lidocaine and prilocaine do not have a cytotoxic effect on gingival epithelial cells and that their use is safe [14]. Accordingly, the cell cytotoxicity and genotoxicity of teething gels containing local anesthesia and different ingredients have not been adequately investigated, and there is a gap in the literature on this subject [8]. Therefore, as there are limited studies on the effects of dental gels on gingival mesenchymal stem cells in the literature, the aim of this study is to examine the cytotoxic effects of dental gels with different contents, which are frequently used during teething, on gingival mesenchymal stem cells.

## Materials-methods

### Human gingival mesenchimal stem cells

Human gingival mesenchimal stem cells (hG-MSCs) were kindly provided by MARSTEM research company (Cell Technologies Industry and Trade Inc., Istanbul, Turkey). Cells were cultivated in a humidified atmosphere in Dulbecco’s Modified Eagle Medium (DMEM, Gibco, Grand Island, USA) supplemented with 10% fetal bovine serum (FBS) and antibiotics at 37 °C and 5% CO_2_. When cells in passage 3 reached to 80% confluency, they were trypsinized and seeded on E-plate.

### Preperation of teething gel solutions

The teething gels used in this study were Dentinox, Gengigel, Osanite, and Jack and Jill. The properties of these gels can be seen in Table 1. Gel solutions at 0.1%, 50% and 80% concentrations were diluted in distilled water and prepared in 3 different concentrations [15].

**Table 1:** Composition of study groups

Materials	Composition	Manufacturer
Dentinox	Lidocaine hydrochloride, Cetylpyridinium chloride	Dendron Brands Limited
Gengigel	Sodyum hyaluronat, Aqua, PEG 400, Xylitol, Polyvinyl alcohol, Cellulose gum, PEG 40 hydrogenated castor oil, PVP, PVM/MA copolymer, VP/Eicosene copolymer, Glyceryl laurate, Carbomer(Polycarbophil), Sodium saccharin, Sodium phosphate, Glycerophosphocholine, Trisodium phosphate, Sodium lactate, Disodium	Ricerfarma, Milano, Italy
Osanite	Chamomilla recutita D6, Calcium phosphoricum D12, Magnesium phosphoricum C6, Calcium carbonicum Hahnemanni C8, Ferrum phosphoricum C8.	Queisser Pharma GmbH & Co. KG, Deutschland
Jack and Jill	Purified water, Glycerin, Hydroxyethyl cellulose, Calendula officinalis extract, Xylitol (Organic), Potassium sorbate, Chamomilla recutita flower extract, Vanilla planifolia flavor (Naturally derived), Citric acid.	Jack & Jill Kids Pty Ltd., London, UK
Complete DMEM (CDMEM) (Control group)	10% FBS (Fetal bovine serum), DMEM (Dulbecco’s Modifed Eagles Medium) supplemented with 1% penicillin/streptomycin	Gibco, Grand Island, USA

### xCELLigence Real-Time Cell Analyzer (RTCA) system

**Fig. 1:**
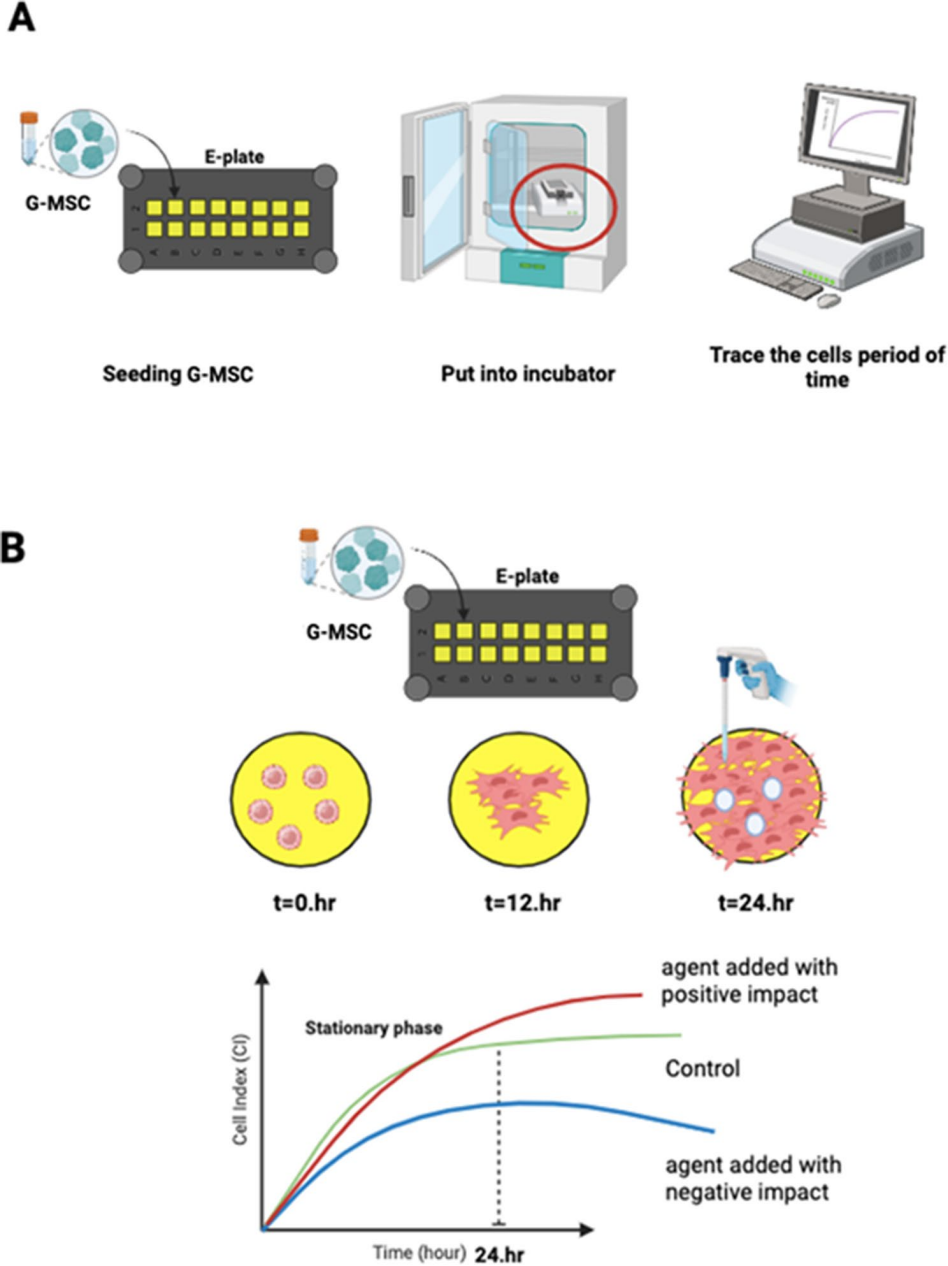
Sequentially, (**A**) General stages of the xCELLigence real-time cell monitoring system. (**B**) Adhesion of G-MSCs in a single well of a gold surface-coated E-plate over time, and the resulting cell tracking graph in this context

The xCELLigence RTCA system (ACEA Biosciences, San Diego, CA, USA) was used exactly as advised by the manufacturer (Fig. 1). In summary, the system operates as follows: This system makes use of three specially designed 16-well E-plate views. These disposable plates are intended for one-time usage only. The plates have gold microelectrodes inserted at the bottom of the wells. In this modified E-Plate, four rows of microelectrode sensors were removed from the middle of each well. This removal enables the use of microscopes to examine cells. 70% of the electrodes are covered at the bottom of the wells. The electrical impedance of these sensor electrodes is measured to follow changes in the cell. Electrical impedance variations are expressed by a unitless metric known as “Cell Index (CI).” When there are no cells in the wells, both the electrode impedance and the CI are 0. After cell seeding, CI will rise. An increase in CI corresponds to an increase in the number of cells that are connected. When additional cells are attached to the surface of the E-Plate, the CI rises. Furthermore, cell viability and cell adhesion strength, rather than cell amount, can influence CI.

### Cell proliferation experiment using xCELLigence RTCA system

Phosphate buffered saline (PBS) (Gibco, Grand Island, NY, USA) rinsed the cells in a T25 flask when they reached 80% confluency. After that, the cells were treated with 0.05% trypsin/EDTA. The flask was filled with 5 mL of full media after 2 min. The cell suspension was centrifuged at 400 x g for 5 min. After resuspending the pellet in 5 mL of medium, the cells were counted using a hemocytometer. In E-Plate view, a standard background was determined before seeding cells by adding 50 L of complete media at 37 °C to wells. After that, in E-plate view, 2*104 cells were planted in each well, and the total volume of wells was adjusted to 200 L with mediaIn a cell culture incubator, the E-plate view was incubated for 30 min. Finally, during 72 h, G-MSCs were monitored every 15 min. After that, measurements were obtained at 0, 30, 60, 90, and 120 h, as well as at 24, 48, and 72 h, and analysis charts were prepared.

### Cytotoxicity experiment using xCELLigence RTCA system

The G-MSC proliferation experiment yielded the ideal cell count for the cytotoxicity experiment; 2*10^4^ cells/well were planted into each well of the E-Plate view. After then, the cells were checked every 30 min. After 24 h, the cells were washed with PBS to remove unattached cells and the medium was replaced; when the cells were in log phase, they were incubated with teething gels. Reproductive behavior of G-MSCs was monitored in real time for 72 h using the xCELLigence RTCA device (ACEA Biosciences). During the study, the proliferation capacity of the cells was expressed with a value called “cell index”.

### Statistical analysis

While evaluating the findings obtained in the study, IBM SPSS Statistics 22 program was used for statistical analysis. The suitability of the parameters for normal distribution was evaluated with Kolmogorov-Smirnov and Shapiro Wilks tests and it was determined that the parameters were suitable for normal distribution. While evaluating the study data, two-way repeated Anova test and post hoc Bonferroni test were used to evaluate the effect of concentration and dental gel on 0-hour and 72-hour viability. Two way ANOVA test and post hoc Tukey HSD test were used to evaluate the effect of concentration and dental gel on cell viability change. Significance was evaluated at *p* < 0.05 level.

## Results

The effect of different teething gels on the viability levels of cells dependent on concentration and time is shown in Table 2. The joint effect of the examined factors on cell viability is statistically significant (*p* = 0.001) (Table 2).

**Table 2:** Evaluation of the effects of time, concentration and teething gel on cell index

Cell Index	Type III Sum of Squares	df	Mean Square	F	p
Time	8.748	1	8.748	12430.44	0.001*
Time * Concentration	3.899	2	1.949	2769.918	0.001*
Time * Gel	4.331	4	1.083	1538.675	0.001*
Time* Concentration * Gel	3.290	8	0.411	584.329	0.001*

Two-way Repeated Measures ANOVA Test *p < 0.05

**Fig. 2:**
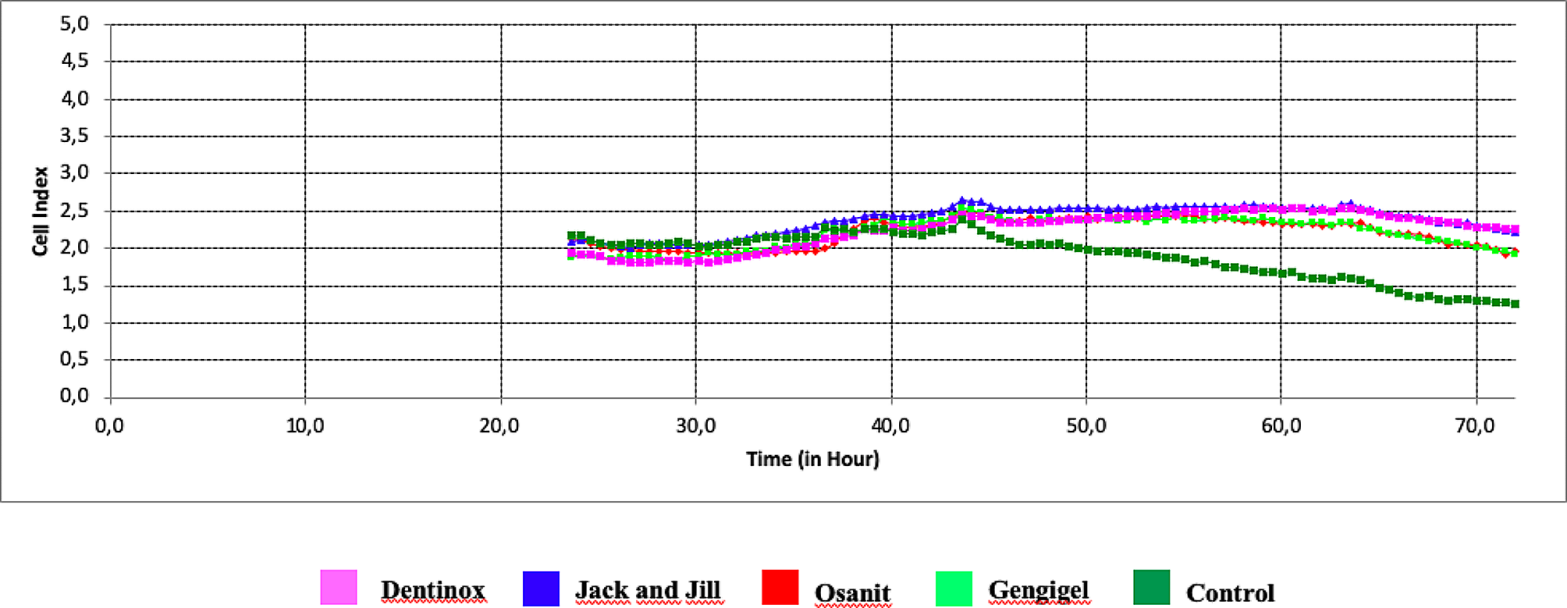
Time-dependent change of cell index (ΔCI) of teething gels at 0.1% concentration

**Fig. 3:**
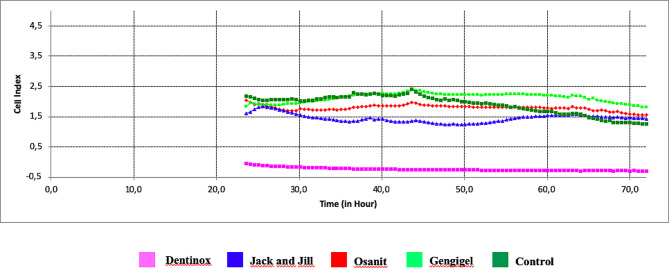
Time-dependent change of cell index (ΔCI) of teething gels at 50% concentration

**Fig. 4:**
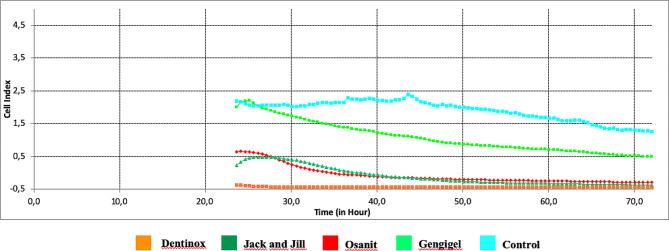
Time-dependent change of cell index (ΔCI) of teething gels at 80% concentration

Time-dependent viability index values of cells exposed to different teething gels and concentrations are shown in Table 3; Figs. 2, 3 and 4. As a result of 72-hour monitoring, the average first “cell index” value of G-MSCs in the control group before the agent was added was 2.17, and at the end of the 72nd hour, this value decreased to 1.26, showing a statistically significant decrease in the viability levels of G-MSCs (*p* = 0.001) (Table 3). While the “cell index” value of the Dentinox group at 0.1% concentration was 1.92 at the time of addition to the culture, it was measured as 2.25 at the end of 72 h. This shows that the presence of Dentinox at 0.1% concentration positively affects the proliferation of G-MSCs and causes a statistically significant increase in the viability levels of the cells (*p* = 0.001). Jack and Jill teething gel at 0.1% concentration causes a statistically significant increase in cell viability levels at the end of the 72nd hour (*p* = 0.001). Gengigel teething gel at 0.1% concentration does not show a statistically significant increase in cell viability levels (*p* = 0.061). The decrease in the “cell index” value of Osanit brand teething gel at 0.1% concentration, which was 2.1 at the time of adding it to the culture, to 1.94 at the end of 72 h, indicates a statistical decrease in the viability level of G-MSCs (*p* = 0.001).

**Table 3:** Comparison of cell index between groups with two-way repeated ANOVA test and change over time for all groups

Concentration	Teething gel	Cell index 0.hour	Cell index 72.hour	
		Mean ± SS	Mean ± SS	p
%0.1	Gengigel	1.898 ± 0.018^a^	1.939 ± 0.024^a^	0.061
	Dentinox	1.925 ± 0.014^b^	2.252 ± 0.001^b^	0.001*
	Osanit	2.113 ± 0.012^c^	1.941 ± 0.012^a^	0.001*
	Jack and Jill	2.100 ± 0.001^c^	2.228 ± 0.012^c^	0.001*
	Control	2.173 ± 0.010^d^	1.260 ± 0.012^d^	0.001*
%0.5	Gengigel	1.898 ± 0.060^a^	1.807 ± 0.002^a^	0.016*
	Dentinox	-0.054 ± 0.013^b^	-0.294 ± 0^b^	0.001*
	Osanit	1.998 ± 0.039^c^	1.561 ± 0.001^c^	0.001*
	Jack and Jill	1.619 ± 0.028^d^	1.427 ± 0.011^d^	0.001*
	Control	2.173 ± 0.010^e^	1.260 ± 0.012^e^	0.001*
%0.8	Gengigel	2.073 ± 0.083^a^	0.493 ± 0.005^a^	0.001*
	Dentinox	-0.372 ± 0.011^b^	-0.444 ± 0.001^b^	0.001*
	Osanit	0.650 ± 0.007^c^	-0.295 ± 0.002^c^	0.001*
	Jack and Jill	0.275 ± 0.059^d^	-0.365 ± 0^d^	0.001*
	Control	2.173 ± 0.010^e^	1.26 ± 0.012^e^	0.001*
p values for teething gels	%0.1	0.001*	0.001*	
	%0.5	0.001*	0.001*	
	%0.8	0.001*	0.001*	
p values for concentration	Gengigel	0.001*	0.001*	
	Dentinox	0.001*	0.001*	
	Osanit	0.001*	0.001*	
	Jack and Jill	0.001*	0.001*	
	Control	1.000	1.000	

Two-way Repeated Measures ANOVA Test, Post hoc Bonferroni test *p < 0.05

*a-b-c-d-e*: There is no difference between the groups with the same

When teething gels prepared at 50% concentration are added to the G-MSC culture, the “cell index” value of G-MSCs to which Dentinox brand gel is added is significantly lower than all other groups (*p* = 0.05). At the end of the 72nd hour, the highest cell index value was observed in Gengigel teething gel at 50% concentration (*p* < 0.05).

It is seen that there is a statistically significant decrease in all cell index values in teething gels at 80% concentration at the end of the 72nd hour (Fig. 1). Cell index values of the cells decreased to - values at this concentration in Dentinox, Osanit, and Jack and Jill teething gels (Fig. 1).

The effects of concentration and teething gels on the change of cell index values are shown in Table 4. There is a statistically significant difference between the concentrations in terms of cell index values at the 72nd hour compared to the 0th hour. (*p* = 0.001). There is a statistically significant difference between the teething gels in terms of cell index change amounts at the 72nd hour compared to the 0th hour (*p*=0.001). The joint effect of concentration and dental gel on the cell index change amounts at the 72nd hour compared to the 0th hour is statistically significant (*p* = 0.001; *p* < 0.05) (Table 4) (Fig. 4).

**Table 4:** Evaluation of the effects of concentration and teething gel on cell index change (ΔCI)

Cell Index	Type III Sum of Squares	df	Mean Square	F	p
Concentration	4.744	2	2.372	1685.315	0.001*
Gel	5.24	4	1.31	930.724	0.001*
Concentration * Gel	7.419	8	0.927	658.907	0.001*

Two-way ANOVA Test *p < 0.05

There is a statistically significant difference in terms of changes in cell index values over time between teething gels at 0.1%, 50% and 80% concentrations (*p* = 0.001) (Table 5).

**Table 5:** Evaluation of the effects of concentration and teething gel on cell index change (ΔCI)

Groups	ΔCI (%0.1)	ΔCI (%0.5)	ΔCI (%0.8)	
	Mean ± SS	Mean ± SS	Mean ± SS	p
Gengigel	0.041 ± 0.032aA	0.091 ± 0.062aA	1.581 ± 0.088aB	0.001*
Dentinox	0.327 ± 0.014bA	0.240 ± 0.013bB	0.072 ± 0.010bC	0.001*
Osanit	0.173 ± 0.024cA	0.437 ± 0.038cB	0.945 ± 0.008cC	0.001*
Jack and Jill	0.128 ± 0.012dA	0.192 ± 0.039cB	0.640 ± 0.059dC	0.001*
Control	0.914 ± 0.002eA	0.914 ± 0.002dA	0.914 ± 0.002cA	1.000
p	0.001*	0.001*	0.001*	

Two-way ANOVA Test, Post hoc Tukey HSD test *p < 0.05

a-b-c-d-e: There is no difference between the groups with the same letter in same column

A-B-C: There is no difference between the groups with the same letter in same line

The time-dependent cell index change of Gengigel, Osanit, and Jack and Jill teething gels at 80% concentration was found to be significantly higher than other concentrations (*p* = 0.001). The amount of cell index change (ΔCI) over time at the 0.1% concentration of Dentinox dental gel is statistically significantly higher than other concentrations (*p* = 0.001) (Table 5).

**Fig. 5:**
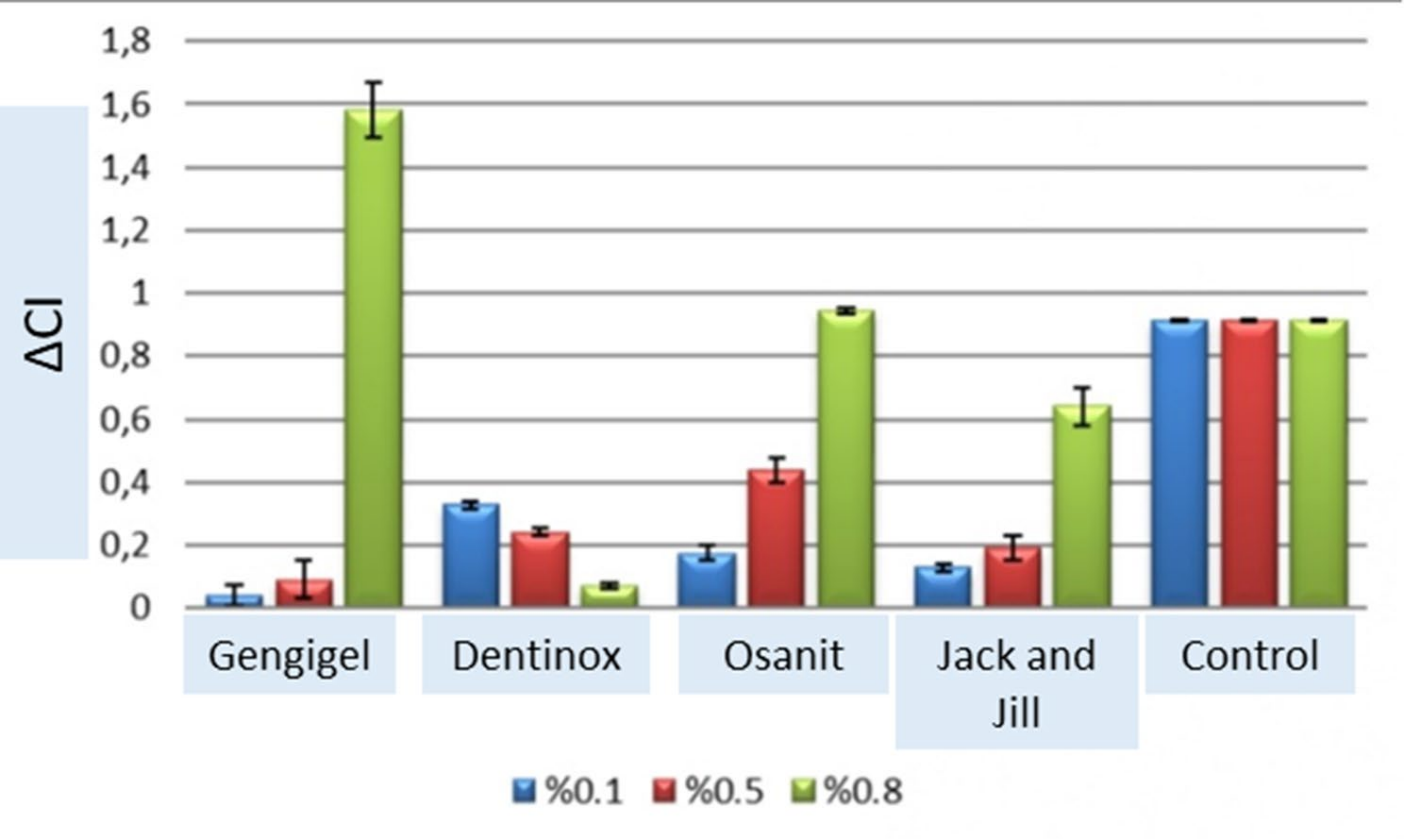
Change of cell index (ΔCI) values depending on time

At 50% concentration, the change in Osanit, Dentinox, and Jack and jill cell index is higher than Gengigel teething gel. At 80% concentration, the highest cell index change belongs to Gengigel teething gel (Fig. 5).

## Discussion

Lidocaine is an amide-type local anesthetic used topically on the oral mucosa for a variety of conditions such as mouth ulcers and teething in children [16]. It provides reversible sensory loss in certain parts of the body by blocking voltage-gated sodium channels, thus preventing the propagation of action potentials along the neuron and the transmission of the pain signal [10].

Besides these properties, the most common symptoms of lidocaine toxicity are central nervous system (CNS) effects presumed to result from selective blockade of inhibitory cortical synapses, including agitation, coma, confusion, hearing loss, respiratory depression, seizures, and visual disturbances [16]. It has been stated in the literature that local anesthetic gels containing lidocaine cause serious adverse reactions such as seizures, respiratory arrest and death [10].

Cell studies have shown that local anesthetics containing lidocaine induce the apoptosis of cells [17–19]. However, lidocaine-containing dental gels carry a high risk of toxicity, methemoglobinemia and central nervous system problems, especially when applied indiscriminately and in excessive amounts to the mucosa of infants and children without the supervision of the healthcare team, since the babies are still developing. For this reason, it has been stated that exogenous hyaluronic acid has recently been used for the treatment of inflammatory conditions in the gums due to the side effects of dental gels containing local anesthesia [20].

Endogenous hyaluronic acid is known to be a high molecular weight glycosaminoglycan of the extracellular matrix that plays a role in growth, infection and repair [21]. It plays an important role in tissue regeneration after inflammation and is stated to facilitate cell migration and differentiation during tissue formation and repair [22]. It is reported that it can be used topically as an anti-inflammatory and antiedema agent [20].

It has been suggested that topical application of a gel called Gengigel containing hyaluronic acid triggers healing in patients with gum infections [23].

Mercan et al. evaluated the effects of solutions containing hyaluronic acid, chlorhexidine and octenidine dhydrochloride on human gingival fibroblasts and reported that the best cell viability was in the group containing hyaluronic acid [24]. Additionally, some studies have shown that high molecular weight hyaluronic acid stimulates cell profiling [25–27].

Therefore, in our study, stem cell viability was examined to shed light on the effects of local anesthetic-containing dental gel, hyaluronic acid-containing dental gel and herbal-containing dental gels on gingival mesenchymal stem cells.

Mesenchymal stem cells contribute to tissue regeneration by supporting the production of factors with anti-inflammatory properties and by differentiating into epithelial-like cells in mucosal infections [15, 28]. It is thought that the anti-inflammatory factor production of mesenchymal stem cells and their contribution to the healing process are important in preventing inflammation in the gums during the teething period, as well as in relieving the symptoms during this period [15]. It is important that the gels used contribute to the healing of the gums during tooth eruption without creating a toxic effect on mesenchymal stem cells. For these reasons, the effects of the teething gels used in our study on gingival mesenchymal stem cells were examined.

Traditional test methods such as MTT and flow cytometry are frequently used to evaluate cell viability. The xCELLigence RTCA system, a new cell viability assessment method used in recent studies along with technological developments, provides label-free detection that allows cells to be tested under more physiological conditions and prevents artifacts that may occur due to use [29–31]. Non-invasive, real-time monitoring of cells allows long-term monitoring of live cells and control of each well simultaneously [32].

In this study, the xCELLigence RTCA system, a new cell viability evaluation method that has advantages over traditional methods, was preferred. This study is the first to examine the effects of teething gels on gingival mesenchymal stem cells.

In our study, it was observed that gel type, concentration and time were effective on cell viability levels. While time-dependent cell index change amounts do not show a statistical difference in the control group depending on concentration, it is observed that the change in cell index value at 80% concentration is statistically higher in the other groups. The highest cell viability when dental gels at 80% concentration were added to the cells was in the control group, followed by Gengigel dental gel containing hyaluronic acid. The lowest cell index value was detected in Dentinox dental gel containing lidocaine. Due to the increase in concentration, it seems that Dentinox teething gel containing lidocaine is the group that most negatively affects the viability of the cells.

As the concentration increases, it is concluded that Gengigel dental gel containing hyaluronic acid has a less toxic effect on cell viability than other gels. These findings support that hyaluronic acid content triggers wound healing and proliferating cell migration.

This study has a number of limitations, such as its incapacity to accurately replicate the in vivo environment due to the lack of saliva and the tissue barriers’ immunological and protective qualities. Furthermore, cytotoxicity was not monitored for durations greater than 72 h. Although this is the first study to compare the cytotoxic effects of various teething gels with the xCELLigence RTCA device, more research is necessary to fully understand these effects.

## Conclusion

As a result, topical dental gels, which are frequently preferred during tooth eruption, however, families should apply these gels in accordance with the instructions of pediatric dentists, paying attention to overdoses.Because it appears that local anesthetic dental gels used in children have a more negative effect on cell viability as concentration increases. It was concluded that the teething gel containing hyaluronic acid, which is stated to have a positive contribution to wound healing, had the most positive effect on cell viability after the control group. More studies are needed to evaluate the effects of teething gels on cells.

## Data Availability

The datasets used and/or analyzed during the current study are available from the corresponding author on reasonable request.

## References

[1] Marks S, Schroeder H (1996). Tooth eruption: theories and facts. Anat Rec.

[2] Tirali E, Erdemci ZY, Çehreli B (2011). Sürme anomalileri. Gazi Üniversitesi Diş Hekimliği Fakültesi Dergisi.

[3] Hernandez JM (2002). Mecanismos Y teorı ´as De La Erupcio ´n Denta- ria.Estado actual. Rev Eur Odont-Estomatol.

[4] Erkut Z, Selmin KÖSE, Dumandağ F (2021). 4–36 ay arası bebeklerin diş çıkarma sürecinde yaşadıkları sorunlar ve annelerin yaptıkları uygulamalar. Dokuz Eylül Üniversitesi Hemşirelik Fakültesi. Elektronik Dergisi.

[5] McIntyre GT, McIntyre GM (2002). Teething Troubles? Br Dent J.

[6] Ispas RS, Mahoney EK, Whyman RA. Teething signs and symptoms: persisting misconceptions among health professionals in New Zealand. NZ Dent J 2013;109(1). 23923149

[7] Sood S, Sood M (2010). Teething: myths and facts. J Clin Pediatr Dentistry.

[8] Topal BG, Yiğit TT, Falay SB (2023). The dentists’attıtudes and knowledge level about teethıng gels. Kocatepe Tıp Dergisi.

[9] Tsang A (2010). Teething, teething pain and teething remedies. Int Dent SA.

[10] Teoh L, Moses GM (2020). Are teething gels safe or even necessary for our children? A review of the safety, efficacy and use of topical lidocaine teething gels. J Paediatr Child Health.

[11] Balit CR, Lynch AM, Gilmore SP, Murray L, Isbister GK (2006). Lidocaine and chlorhexidine toxicity in children resulting from mouth paint ingestion: a bottling problem. J Paediatr Child Health.

[12] FDA Drug Safety Communication [04-07-2011]. Reports of a rare, but serious and potentially fatal adverse effect with the use of over-the-counter (OTC) benzocaine gels and liquids applied to the gums or mouth. https://www.fda.gov/drugs/drug-safety-and-availability/fda-drug-safety-communication-reports-rare-serious-and-potentially-fatal-adverse-effect-use-over,Accessed on 18.01.2022.

[13] Fedder C, Beck-Schimmer B, Aguirre J, Hasler M, Roth-Z’graggen B, Urner M (2010). In vitro exposure of human fibroblasts to local anaesthetics impairs cell growth. Clin Experimental Immunolog.

[14] Khongkhunthian S, Supanchart C, Yotsawimonwat S, Okonogi S (2017). In vitro oral epithelium cytotoxicity and in vivo inflammatory inducing effects of anesthetic rice gel. Drug Discoveries Ther.

[15] Birant S, Duran Y, Gokalp M, Akkoc T, Seymen F (2021). Effects of different detergent-containing children’s toothpastes on the viability, osteogenic and chondrogenic differentiation of human dental periodontal ligament stem cells and gingival stem cells in vitro. Tissue Cell.

[16] Curtis LA, Dolan TS, Seibert HE (2009). Are one or two dangerous? Lidocaine and topical anesthetic exposures in children. J Emerg Med.

[17] Dregalla RC, Lyons NF, Reischling PD, Centeno CJ (2014). Amide-type local anesthetics and human mesenchymal stem cells: clinical implications for stem cell therapy. Stem Cells Transl Med.

[18] Boselli E, Duflo F, Debon R, Allaouchiche B, Chassard D, Thomas L, Portoukalian J (2003). The induction of apoptosis by local anesthetics: a comparison between lidocaine and ropivacaine. Anesth Analg.

[19] Oliveira AC, Rodríguez IÁ, Garzón I, Martín-Piedra MÁ, Alfonso-Rodríguez CA, García JM (2014). An early and late cytotoxicity evaluation of lidocaine on human oral mucosa fibroblasts. Experimental Biology Med.

[20] Yashika J (2013). Clinical evaluation of 0.2% hyaluronic acid containing gel in the treatment of gingivitis. Med J Dr D Y Patil Univ.

[21] Moseley R, Waddington RJ, Embery G (2002). Hyaluronan and its potential role in periodontal healing. Dent Update.

[22] Oksala O, Salo T, Tammi R, Häkkinen LARI, Jalkanen M, Inki P, Larjava H (1995). Expression of proteoglycans and hyaluronan during wound healing. J Histochem Cytochemistry.

[23] Suresh DK, Vandana KL, Mehta DS (2001). Intracrevicular application of 0.3% flurbiprofen gel and 0.3% triclosan gel as anti inflammatory agent. A comparative clinical study. Indian J Dent Res.

[24] Mercan U, Gonen ZB, Salkin H, Ulker GMY, Meral DG (2019). Comparison of the effect of postoperative care agents on human gingival fibroblasts: a preliminary study. Eur Oral Res.

[25] Yoneda M, Yamagata M, Suzuki S, Kimata K (1988). Hyaluronic acid modulates proliferation of mouse dermal fibroblasts in culture. J Cell Sci.

[26] Kawasaki K, Ochi M, Uchio Y, Adachi N, Matsusaki M (1999). Hyaluronic acid enhances proliferation and chondroitin sulfate synthesis in cultured chondrocytes embedded in collagen gels. J Cell Physiol.

[27] Ahrens T, Assmann V, Fieber C, Termeer C, Herrlich P, Hofmann M, Simon JC (2001). CD44 is the principal mediator of hyaluronic-acid-induced melanoma cell proliferation. J Invest Dermatol.

[28] Zhang QZ, Nguyen AL, Yu WH, Le AD (2012). Human oral mucosa and gingiva: a unique reservoir for mesenchymal stem cells. J Dent Res.

[29] Yavuz SA, Sürmelioğlu D (2020). Evaluation of cytotoxicity of different Universal Bonds using the Xcelligence System. Cumhuriyet Dent J.

[30] Urcan E, Haertel U, Styllou M, Hickel R, Scherthan H, Reichl FX (2010). Real-time xCELLigence impedance analysis of the cytotoxicity of dental composite components on human gingival fibroblasts. Dent Mater.

[31] Demirel G, Gür G, Demirsoy FF, Altuntaş EG, Yener-Ilce B, Kiliçarslan MA (2020). Cytotoxic effects of contemporary bulk-fill dental composites: a real-time cell analysis. Dent Mater J.

[32] Özdemir A, Ark M. xCELLigence Real Time Cell Analysis System A New Method for cell proliferation and cytotoxicity. Niche J 2014;2(2).

